# Comparison of surgical and radiotherapy outcomes in octogenarians with early-stage non-small cell lung cancer: a SEER database retrospective cohort study

**DOI:** 10.1007/s40520-025-02948-2

**Published:** 2025-02-27

**Authors:** Wenxuan Hua, Zhigang Zhang, Lianfang Ni, Xinmin Liu

**Affiliations:** Department of Geriatrics, Peking University First Hospital, Beijing, 100034 China

**Keywords:** NSCLC, Octogenarians, Treatment, SEER database, Survival, Propensity score matching

## Abstract

**Background:**

Lung cancer remains the leading cause of death worldwide, yet optimal treatment strategies for octogenarians with early-stage non-small cell lung cancer (NSCLC) remain unclear.

**Aims:**

To investigate treatment patterns and survival outcomes in octogenarians and older with early-stage NSCLC.

**Methods:**

A retrospective cohort study was conducted using Surveillance, Epidemiology, and End Results database. Patients aged ≥ 80 years with stage I–IIA NSCLC diagnosed between 2011 and 2020 were included. Primary treatments included surgery, radiation, and no treatment. Kaplan–Meier curves were used to evaluate overall survival (OS) and cancer-specific survival (CSS) stratified by treatment and year. Propensity score matching balanced clinical characteristics between surgery and radiation groups, followed by Cox regression analysis. Survival outcomes were further compared within matched subgroups stratified by tumor size.

**Results:**

Among 7,372 patients, median survival was 67 months for surgery and 33 months for radiotherapy. Radiotherapy use increased from 31.2% in 2011 to 49.4% in 2020, while surgery rates declined. Multivariate analysis (*N* = 2,434) showed radiotherapy was associated with worse OS (hazard ratio = 1.96, 95% CI = 1.78–2.15, *P* < 0.001) compared to surgery.

**Discussion:**

Radiotherapy is increasingly used to treat early-stage NSCLC in octogenarians, yet surgery provides superior long-term survival. Limitations of lack of detailed comorbidity data and differentiation between conventional radiotherapy and stereotactic ablative radiotherapy (SABR) may have expanded the advantages of surgery. Meanwhile, patient performance status and preferences must be considered in treatment decisions.

**Conclusions:**

Surgery remains the preferred treatment option for eligible octogenarians with early-stage NSCLC.

**Supplementary Information:**

The online version contains supplementary material available at 10.1007/s40520-025-02948-2.

## Introduction

Lung cancer is by far the leading cause of death in both men and women in the United States [1]. In 2024, an estimated 234,580 new cases (116,310 in males and 118,270 in females) of lung and bronchial cancer will be diagnosed, and 125,070 deaths (65,790 in males and 59,280 in females) are estimated to occur because of the disease [1]. One of the most rapidly growing populations with lung cancer is patients older than 80 years, who accounted for 25.7% of all lung cancer deaths (34,900/134,504) in 2021 due to the aging of the general population. Non–small cell lung cancer (NSCLC) accounts for approximately 80% of all lung cancers, whereas small-cell lung cancer (SCLC) represents approximately 14% of lung cancer cases [2]. According to Ganti’s cross-sectional epidemiological analysis, due to the efficacy of the implementation of annual screening programs from 2010 to 2017, the incidence of stage II and III NSCLC was stable, and that of stage IV decreased slightly from 21.7 to 19.6%, whereas the incidence of stage I increased from 10.8–13.2% [3]. Therefore, it is important to determine the best treatment strategies for octogenarians with early-stage NSCLC.

For early-stage NSCLC (stage I–II, N0), surgical resection remains the primary and preferred local treatment modality [4], whereas octogenarians and older individuals are often associated with multiple comorbidities, poor cardiopulmonary function, frailty, and increased operative risk, which complicates lung cancer treatment. Definitive radiotherapy (RT), preferably stereotactic ablative radiotherapy (SABR), is recommended for patients with early-stage NSCLC who are medically inoperable or those who refuse surgery [5–10]. Over the last decade, RT has taken the place of surgery as the prevailing treatment modality for octogenarians with early-stage NSCLC [11–13]. However, whether surgery or radiotherapy exerts a superior impact on the treatment of such patients remains uncertain. Conducting randomized clinical trials in octogenarians is challenging. This study utilized the Surveillance, Epidemiology and End Results (SEER) database to compare survival differences between octogenarians and older patients who underwent surgery versus those who opted for radiotherapy in early-stage NSCLC patients.

## Methods

We conducted a retrospective cohort study utilizing the SEER 17 Registries database (2000–2020). Given the retrospective nature of this study and the anonymization of patient data, institutional review and informed consent were not necessary for this investigation. The study adheres to the STROBE guidelines for the reporting of observational studies.

### Data sources

The SEER 17 Registries database (2000–2020) released in November 2022, which covers approximately 26.5% of the U.S. population, provides representative patient-level cancer data from 17 geographically diverse populations. The follow-up of the patients concluded by December 31, 2020. The SEER database was analyzed via SEER Stat (version 8.4.1; http://www.seer.cancer. gov).

### Patient selection

Patients aged 80 years or older who were diagnosed with their first primary early-stage NSCLC from January 2011 to December 2020 were included. The early stage was defined as clinical or pathologic T1–T2N0M0 (T ≤ 5 cm) classified as stage I–IIA according to the 8th Edition of the American Joint Committee on Cancer (AJCC) Staging Manual. Patients diagnosed with NSCLC were included using histology and topography codes from the International Classification of Diseases for Oncology, Third Edition (ICD-O-3). Exclusion criteria included diagnosis by autopsy/death certificate only, tumor size > 5 cm, and incomplete data on patient demographics or survival time.

### Variables

The primary outcomes of this study were overall survival (OS) and cancer-specific survival (CSS). Covariates included patient demographics (age, sex, race, median household income and geographic location), tumor characteristics (size, location, grade, and histologic type) and treatment details (radiotherapy, surgery and chemotherapy). The treatment categories included surgical intervention, radiotherapy, chemotherapy, and opting for no intervention. The SEER database did not differentiate between conventional radiotherapy and SABR, potentially leading to an underestimation of SABR’s effectiveness compared to surgery. Currently, there are no guidelines recommending the concurrent use of radiotherapy and surgery or the use of chemotherapy in the treatment of early lung cancer. Therefore, we excluded patients who received both surgery and radiotherapy or chemotherapy from the survival analysis. The surgical types included lobectomy, segmentectomy, wedge resection and others (e.g., pneumonectomy).

### Statistical analysis

Categorical variables were presented as counts and percentages and compared using the Pearson χ² test. A total of 5,496 patients, excluding those diagnosed with NSCLC after 2018 or those who received concurrent surgery, radiotherapy, or chemotherapy, were included in the survival analysis. Survival outcomes were estimated using the Kaplan-Meier method, stratified by treatment type and year of diagnosis, and compared with the log-rank test.

To adjust for baseline differences in demographic and clinical characteristics, 1:1 propensity score matching (PSM) was performed between the surgery and radiation groups. Matching variables included age, sex, year of diagnosis, race, median household income, geographic location, tumor grade, tumor size, and histology. Patients were matched without replacement, ensuring each was matched only once. Covariate balance was assessed using the cobalt package, with standardized mean differences (SMDs) < 0.1 indicating acceptable balance. Missing data for tumor grade were addressed by excluding patients with missing values for this variable. Before matching, the surgery group included 2,065 patients and the radiation group 1,217 patients. After matching, 2,434 patients (1,217 from each group) were successfully paired.

Following PSM, univariable and multivariable Cox proportional hazards regression models were used to compare the effectiveness of surgery versus radiation on OS. The multivariable model was constructed by selecting covariates based on clinical relevance and statistical significance (*P* < 0.05) from the univariate analysis. Hazard ratios (HRs) with 95% confidence intervals (CIs) were estimated. The proportional hazards assumption was tested using Schoenfeld residuals, with no violations detected (*P* > 0.05).

A key limitation of this analysis was the lack of detailed information on patient performance status and comorbidities in the SEER database, which limited full adjustment for potential confounders. To explore this, a Kaplan-Meier analysis was conducted comparing survival outcomes in matched patients who were recommended for surgery but opted for radiotherapy. Sensitivity analyses were also performed by stratifying patients by tumor size to assess differences between surgery and radiation.

All statistical tests were two-sided, with a significance threshold of *P* < 0.05. Statistical analyses were conducted using R software (version 4.2.3; The R Project for Statistical Computing).

## Results

### Patient characteristics

A total of 31,371 patients aged 80 years or older were diagnosed with NSCLC in the SEER database from 2011 to 2020, including 8042 patients with early-stage I–IIA (AJCC 8th) NSCLC, 7372 of whom met the study entry criteria. The patient characteristics by the year of cancer diagnosis are summarized in Table 1. In total, 3085 patients (41.8%) were male and 6705 patients (91.0%) were diagnosed between the ages of 80 and 90. The median follow-up time was 39 months [95% CI, 38–41 months].

**Table 1:** Patient characteristics

	2011–2012	2013–2014	2015–2016	2017–2018	2019–2020	Overall	*P* value
	(*N* = 1287)	(*N* = 1344)	(*N* = 1482)	(*N* = 1646)	(*N* = 1613)	(*N* = 7372)	
**Gender**							0.502
Male	537 (41.7%)	558 (41.5%)	639 (43.1%)	662 (40.2%)	689 (42.7%)	3085 (41.8%)	
Female	750 (58.3%)	786 (58.5%)	843 (56.9%)	984 (59.8%)	924 (57.3%)	4287 (58.2%)	
**Age**							0.268
80–89	1168 (90.8%)	1242 (92.4%)	1351 (91.2%)	1489 (90.5%)	1455 (90.2%)	6705 (91.0%)	
≥90	119 (9.2%)	102 (7.6%)	131 (8.8%)	157 (9.5%)	158 (9.8%)	667 (9.0%)	
**Race**							0.004
White	1116 (86.7%)	1157 (86.1%)	1253 (84.5%)	1377 (83.7%)	1331 (82.5%)	6234 (84.6%)	
Black	62 (4.8%)	62 (4.6%)	95 (6.4%)	99 (6.0%)	101 (6.3%)	419 (5.7%)	
Asian or Pacific Islander	102 (7.9%)	115 (8.6%)	126 (8.5%)	154 (9.4%)	160 (9.9%)	657 (8.9%)	
Others	7 (0.6%)	10 (0.7%)	8 (0.6%)	16 (1.0%)	21 (1.3%)	62 (0.9%)	
**Rural-Urban**							0.621
Urban	1163 (90.4%)	1197 (89.1%)	1326 (89.5%)	1471 (89.4%)	1460 (90.5%)	6617 (89.8%)	
Rural	124 (9.6%)	147 (10.9%)	156 (10.5%)	175 (10.6%)	153 (9.5%)	755 (10.2%)	
**Median household income**							< 0.001
$75,000+	425 (33.0%)	505 (37.6%)	594 (40.1%)	814 (49.5%)	1042 (64.6%)	3380 (45.8%)	
<$75,000	862 (67.0%)	839 (62.4%)	888 (59.9%)	832 (50.5%)	571 (35.4%)	3992 (54.2%)	
**Histology**							< 0.001
ACA	733 (57.0%)	777 (57.8%)	912 (61.5%)	1025 (62.3%)	1045 (64.8%)	4492 (60.9%)	
SCC	400 (31.1%)	435 (32.4%)	444 (30.0%)	486 (29.5%)	460 (28.5%)	2225 (30.2%)	
Other	154 (12.0%)	132 (9.8%)	126 (8.5%)	135 (8.2%)	108 (6.7%)	655 (8.9%)	
**Grade**							< 0.001
Grade I	216 (16.8%)	223 (16.6%)	226 (15.2%)	254 (15.4%)	239 (14.8%)	1158 (15.7%)	
Grade II	436 (33.9%)	399 (29.7%)	481 (32.5%)	489 (29.7%)	466 (28.9%)	2271 (30.8%)	
Grade III	328 (25.5%)	332 (24.7%)	316 (21.3%)	287 (17.4%)	276 (17.1%)	1539 (20.9%)	
Grade IV	18 (1.4%)	12 (0.9%)	13 (0.9%)	2 (0.1%)	4 (0.2%)	49 (0.7%)	
Unknown	289 (22.5%)	378 (28.1%)	446 (30.1%)	614 (37.3%)	628 (38.9%)	2355 (31.9%)	
**Laterality**							0.104
Right	535 (41.6%)	584 (43.5%)	609 (41.1%)	679 (41.3%)	689 (42.7%)	3096 (42.0%)	
Left	752 (58.4%)	760 (56.5%)	873 (58.9%)	967 (58.7%)	924 (57.3%)	4276 (58.0%)	
**Tumor size**							0.232
0–1 cm	47 (3.7%)	45 (3.3%)	64 (4.3%)	81 (4.9%)	76 (4.7%)	313 (4.2%)	
1–2 cm	358 (27.8%)	390 (29.0%)	429 (28.9%)	514 (31.2%)	509 (31.6%)	2200 (29.8%)	
2–3 cm	455 (35.4%)	452 (33.6%)	504 (34.0%)	536 (32.6%)	521 (32.3%)	2468 (33.5%)	
3–4 cm	272 (21.1%)	291 (21.7%)	294 (19.8%)	340 (20.7%)	328 (20.3%)	1525 (20.7%)	
4–5 cm	155 (12.0%)	166 (12.4%)	191 (12.9%)	175 (10.6%)	179 (11.1%)	866 (11.7%)	
**Treatment**							0.09
None	250 (19.4%)	220 (16.4%)	223 (15.0%)	283 (17.2%)	293 (18.2%)	1269 (17.2%)	
Treatment	1037 (80.6%)	1124 (83.6%)	1259 (85.0%)	1363 (82.8%)	1320 (81.8%)	6103 (82.8%)	
**Radiation**							< 0.001
Yes	446 (34.7%)	556 (41.4%)	671 (45.3%)	798 (48.5%)	787 (48.8%)	3258 (44.2%)	
No	841 (65.3%)	788 (58.6%)	811 (54.7%)	848 (51.5%)	826 (51.2%)	4114 (55.8%)	
**Surgery**							< 0.001
Yes	600 (46.6%)	572 (42.6%)	591 (39.9%)	559 (34.0%)	537 (33.3%)	2859 (38.8%)	
No	687 (53.4%)	772 (57.4%)	891 (60.1%)	1087 (66.0%)	1076 (66.7%)	4513 (61.2%)	
**Chemotherapy**							0.118
Yes	58 (4.5%)	45 (3.3%)	42 (2.8%)	61 (3.7%)	69 (4.3%)	275 (3.7%)	
No	1229 (95.5%)	1299 (96.7%)	1440 (97.2%)	1585 (96.3%)	1544 (95.7%)	7097 (96.3%)	
**Single treatment**	974(75.7%)	1076(80.1%)	1214(81.9%)	1309(79.5%)	1251(77.6%)	5824(79.0%)	< 0.001
Surgery only	567 (44.1%)	547 (40.7%)	575 (38.8%)	543 (33.0%)	498 (30.9%)	2730(37.0%)	
Radiation only	389 (30.2%)	517 (38.5%)	631 (42.6%)	751 (45.6%)	744 (46.1%)	3032 (41.1%)	
Chemotherapy only	18 (1.4%)	12 (0.9%)	8 (0.5%)	15 (0.9%)	9 (0.6%)	62 (0.8%)	
**Multiple treatments**							
More than 1 treatment	63(4.9%)	48(3.6%)	45(3.0%)	54(3.3%)	69(4.3%)	279(3.8%)	0.06
Surgery and radiation	27 (2.1%)	16 (1.2%)	11 (0.7%)	9 (0.5%)	13 (0.8%)	76 (1.0%)	< 0.001

### Temporal trend analysis

**Fig. 1:**
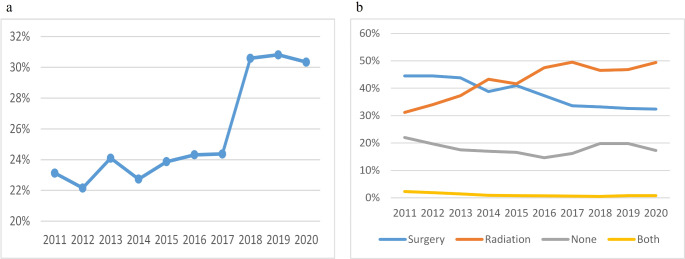
Early-stage NSCLC in octogenarians and older: proportion and treatment pattern

The total number of NSCLC patients aged 80 and older remained relatively stable, from 3,083 in 2011 to 3,346 in 2019. While the proportion of early-stage NSCLC patients increased (Fig. 1a). The number of patients diagnosed with ACA gradually increased annually, from 376 patients (57.8%) in 2011 to 452 patients (63.1%) in 2020.

Figure 1a illustrated the proportion of early-stage NSCLC among octogenarians and older compared to all cases. The percentage of early-stage NSCLC patients was higher (over 30%) during 2018–2020 compared to the earlier period from 2011 to 2017 (less than 25%). Figure 1b showed the distribution of initial treatments (surgery, radiation, both surgery and radiation, and none) for early-stage octogenarians and older.

### Treatment and survival of the entire cohort

#### Treatment pattern in early-stage octogenarians

The initial treatment for early-stage octogenarians is shown in Table 1. There was a notable increase in the proportion of octogenarians who underwent radiotherapy, increasing from 31.2% in 2011 to 49.4% in 2020 (Fig. 1b). Concurrently, there was a decrease in the percentage of patients treated with surgery, decreasing from 44.5 to 32.4%. Since 2014, radiotherapy has surpassed surgery, emerging as the predominant treatment for octogenarians. The percentage of patients who received neither radiotherapy nor surgery decreased from 22 to 17.3% (*P* = 0.09). In addition, tumor size influences the treatment modality (Table 2). As the tumor size increased, the number of patients who underwent surgery decreased, while the number of patients who did not undergo surgery or radiotherapy increased. Within the surgery group, there was a tendency to opt for lobectomy as the tumor size increased.

**Table 2:** Treatments stratified by tumor size

Size(cm)	0–1	1–2	2–3	3–4	4–5	Overall
Surgery	176 (58.7%)	965 (44.9%)	878 (36.9%)	491 (34.6%)	220 (28.2%)	2730 (38.8%)
Lobectomy	77 (43.8%)	521 (54.0%)	618 (70.4%)	393 (80.0%)	186 (84.5%)	1795 (65.8%)
Segmentectomy	20 (11.4%)	107 (11.1%)	60 (6.8%)	19 (3.9%)	5 (2.3%)	211 (7.7%)
Wedge resection	74 (42.0%)	303 (31.4%)	173 (19.7%)	61 (12.4%)	16 (7.3%)	627 (23.0%)
Other	5 (2.8%)	34 (3.5%)	27 (3.1%)	18 (3.7%)	13 (5.9%)	97 (3.6%)
Radiation	88 (29.3%)	893 (41.5%)	1112 (46.7%)	622 (43.8%)	317 (40.6%)	3032 (43.1%)
No treatment	36 (12.0%)	292 (13.6%)	392 (16.5%)	306 (21.6%)	243 (31.2%)	1269 (18.0%)

### Overall survival and cancer-specific survival of different treatment during 2011–2020

**Fig. 2:**
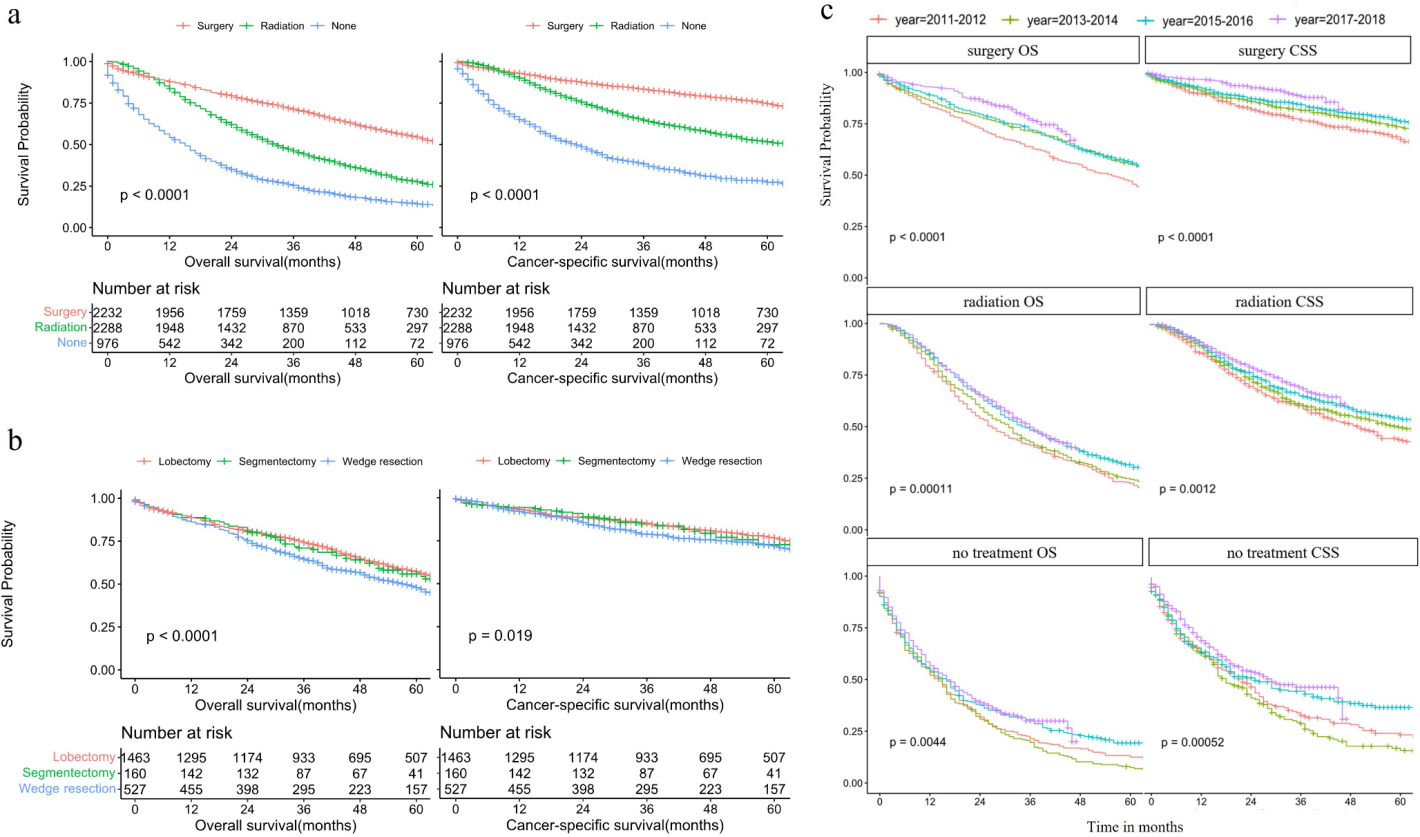
Overall survival and cancer-specific survival curves of patients receiving different treatments from 2011–2020 (*N* = 5,496)

Excluding patients diagnosed with NSCLC after 2018 and those who received concurrent surgery, radiotherapy, or chemotherapy, 5,496 patients remained eligible for the survival analysis. The overall 3-year survival of the entire cohort was 52.54% (95% confidence interval [CI], 51.28-53.83%). Patients receiving surgery had better survival compared with patients receiving radiotherapy (median OS (mOS) 67 months [range, 63–70] vs. mOS 33 months [range, 31–35]). Patients who did not undergo surgery or radiotherapy had the poorest OS (mOS 15 months [range, 13–16]). The OS and CSS curves for different treatments are illustrated in Fig. 2. During the initial seven months, the surgery group exhibited a worse OS than did the radiation group, which was attributed to a greater incidence of perioperative mortality. Among surgical approaches, lobectomy remains the predominant method, followed by wedge resection and segmentectomy. The 1-year OS did not exhibit significant variation between lobectomy and other procedures, but long-term OS indicated a superior outcome for lobectomy (lobectomy, mOS 72 months [range, 68–77] vs. segmentectomy, mOS 71 months [range, 57–105] vs. wedge resection, mOS 58 months [range, 51–65]). The 3-year OS improved in surgery, radiotherapy and no treatment, from 63.85%, 41.01% and 21.41%, respectively, in 2011 to 77.46%, 49.70% and 30.00%, respectively, in 2018 (Fig. 2).

### Univariate and multivariate analysis for overall survival

A 1:1 propensity score matching (PSM) analysis was conducted to minimize bias between the surgery and radiation groups. Prior to matching, the surgery group included 2,065 patients and the radiation group 1,217 patients. After matching, 2,434 patients were successfully paired, resulting in well-balanced cohorts (eTable 1). Univariate analysis identified significant associations with overall survival (OS) for variables including age at diagnosis, sex, year of diagnosis, race, geographic location, median household income, tumor grade, tumor size, histology, and treatment modality (*P* < 0.05) (Table 3). In the multivariate model, younger age, female sex, later year of diagnosis, Asian or Pacific Islander race, lower tumor grade, smaller tumor size, non-ACA or non-SCC histology, and surgical treatment were independent factors associated with improved OS. Patients treated with radiation had significantly worse OS outcomes compared to those treated with surgery (HR = 1.96, 95% CI = 1.78–2.15, *P* < 0.001).

**Table 3:** Univariate and Multivariate Analysis of OS in Matched Cohort (*N* = 2,434)

Variable	Univariate analysis		Multivariate analysis	
	HR (95%CI)	*P* value	HR (95%CI)	*P* value
Ages of diagnosis	1.37(1.16–1.63)	**0.001**	1.21 (1.02–1.44)	**0.030**
Gender	0.77(0.71–0.82)	**< 0.001**	0.83 (0.78–0.87)	**< 0.001**
Years of diagnosis				
2011–2012	1			
2013–2014	0.85(0.74–0.97)	**0.020**	0.89 (0.78–1.02)	0.097
2015–2016	0.81(0.7–0.93)	**0.003**	0.86 (0.74–0.99)	**0.041**
2017–2018	0.67(0.56–0.78)	**< 0.001**	0.73 (0.62–0.85)	**< 0.001**
Race				
White	1		1	
Black	0.98(0.78–1.23)	0.874	1.01 (0.81–1.26)	0.917
Asian or Pacific Islander	0.79(0.64–0.97)	**0.025**	0.81 (0.66–0.99)	**0.050**
Others	1.05(0.53–2.11)	0.883	0.94 (0.47–1.86)	0.859
Rural-Urban	1.22(1.04–1.42)	**0.013**	1.02 (0.86–1.20)	0.847
Median household income	1.12(1.01–1.25)	**0.033**	1.02 (0.91–1.14)	0.754
Laterality	0.96(0.87–1.06)	0.432		
Grade				
Grade I	1		1	
Grade II	1.94(1.35–2.79)	**< 0.001**	1.90 (1.33–2.69)	**0.001**
Grade III	0.95(0.73–1.26)	0.742	1.05 (0.79–1.40)	0.716
Grade IV	1.10(0.96–1.27)	0.177	1.16 (1.01–1.34)	**0.038**
Tumor size(cm)				
0–1	1		1	
1–2	2.11(1.3–3.44)	**0.003**	1.84 (1.13–3.00)	**0.014**
2–3	2.49(1.53–4.03)	**< 0.001**	2.12 (1.31–3.43)	**0.002**
3–4	2.97(1.83–4.83)	**< 0.001**	2.55 (1.57–4.14)	**< 0.001**
4–5	3.77(2.3–6.18)	**< 0.001**	3.23 (1.98–5.28)	**< 0.001**
Histology				
ACA	1		1	
SCC	1.08(0.92–1.26)	0.352	0.92 (0.79–1.07)	0.316
Other	0.77(0.69–0.86)	**< 0.001**	0.85 (0.75–0.96)	**0.005**
Treatment				
Surgery	1		1	
Radiation	1.90(1.71–2.11)	**< 0.001**	1.96 (1.78–2.15)	**< 0.001**

### Subgroup analysis

#### Survival of patients who received surgery versus radiotherapy (surgery recommended but refused)

PSM with a 1:1 ratio for age, gender, years of diagnosis, race, grade, tumor size and histology ensured balanced variables in surgery and radiotherapy groups (surgery recommended but refused). After PSM, with 174 patients in each group, surgery showed significantly better OS (HR = 1.57, 95% CI = 1.28–1.93, *P* < 0.001) and CSS ((HR = 1.74, 95% CI = 1.30–2.32, *P* < 0.001) compared to radiotherapy.

#### Survival of patients who received surgery without lymph node examination versus radiotherapy

PSM with a 1:3 ratio for age, gender, years of diagnosis, race, grade, tumor size and histology ensured balanced variables in surgery without lymph node examination (LNE) (420 patients) and radiotherapy (1217 patients). Surgery again resulted in better OS (HR = 1.65, 95% CI = 1.44–1.91, *P* < 0.001) and CSS (HR = 1.62, 95% CI = 1.33–1.98, *P* < 0.001).

#### Comparison of patients who received surgery and radiotherapy stratified by tumor size

We stratified patients by tumor size (0–1 cm, 1–2 cm, 2–3 cm, 3–4 cm, and 4–5 cm) and compared OS and CSS between the surgery and radiotherapy groups. Except for the 0–1 cm group, where there was no difference in CSS between the surgery and radiotherapy groups, surgery resulted in longer OS and CSS than radiotherapy in all other size categories (eFig. 1 and eTable 2).

Further analysis in matched subgroups of surgery without lymph node evaluation (LNE) versus radiotherapy within each size category showed that surgery without LNE provided better OS and CSS in all groups, except for the 0–1 cm category (eTable 3).

Finally, survival analysis was performed in matched subgroups of surgery and radiotherapy (where surgery was recommended but refused) within each size category. The sample size in the 0–1 cm group was too small to yield meaningful results. In the 1–2 cm and 4–5 cm groups, no significant differences were found in OS or CSS between surgery and radiotherapy. However, in the 2–3 cm and 3–4 cm groups, surgery outperformed radiotherapy in both OS and CSS (eTable 4).

## Discussion

The current study highlights a significant increase in the proportion of early-stage NSCLC patients during 2018–2020, surpassing 30% of total cases, compared to less than 25% observed during 2011–2017. This upward trend suggests advancements in lung cancer screening and early detection, possibly influenced by increased health consciousness and enhanced healthcare practices. In light of the rapid progress in surgical and radiation techniques, we aimed to analyze the evolving treatment strategies and survival outcomes among octogenarians with early-stage NSCLC.

The use of definitive radiotherapy (RT) in early-stage NSCLC among octogenarians and older individuals increased from 31.2% in 2011 to 49.4% in 2020. This percentage surpassed that of surgery, which decreased from 44.5% in 2011 to 32.4% in 2020; RT has been the primary treatment method since 2014. Moreover, the proportion of patients managed with observation decreased from 22 to 17.3%. Tumor size influences treatment choice, with larger tumors leaning toward RT or not receiving active treatment, leading to a decrease in surgical interventions. Among surgical methods, larger tumor diameters tend to favor lobectomy. This trend underscores the evolving landscape of treatment preferences in older patients, with radiotherapy techniques like SABR becoming increasingly popular. SABR is particularly well-suited for elderly patients, offering high-dose radiation to tumors while minimizing exposure to surrounding healthy tissues. Compared with conventional fractionated RT, SABR employs brief sessions of highly precise and dose-intensive (ablative) radiation therapy that is precisely administered to small targets and has achieved superior primary tumor control and overall survival [10]. A real-world study by Van Rossum et al. [14] at the Netherlands Cancer Institute, involving 7,279 patients (mean age 72.5 years, 21.6% aged over 80), found that SABR for stage I NSCLC resulted in low acute toxicity and an acceptable 90-day mortality, even among octogenarians. The study revealed that advanced age did not increase acute toxicity or mortality risk, positioning SABR as a viable option for elderly patients. Its non-invasive nature, brief treatment schedule, and favorable safety profile have driven its increasing use in clinical practice.

In our study, patients who underwent surgery had a median survival of 67 months and a 3-year OS of 71%. The long-term OS of patients who underwent RT was inferior to that of patients who underwent surgery, with a median survival of 33 months and a 3-year OS of 46%. Those who did not receive either surgery or radiotherapy had the lowest overall survival, with a median survival of 15 months and a 3-year OS of 25%. To mitigate potential biases, we conducted PSM to align the surgery and RT groups as closely as possible. However, the lack of comprehensive data regarding patient performance status and comorbid conditions in the SEER database limits our ability to achieve complete matching. This limitation was underscored by our further comparison of outcomes between matched patients who were recommended for surgery but who refused and opted for radiotherapy instead. The surgery group consistently maintained significantly superior OS (HR = 1.57, 95% CI = 1.28–1.93) and CSS (HR = 1.74, 95% CI = 1.30–2.32) compared to the radiotherapy group of patients who were recommended surgery but ultimately declined (both *P* < 0.001). Given these findings, we recommend that octogenarians and older individuals, provided they can tolerate surgery, prioritize surgical intervention. This recommendation aligns with studies comparing surgery and SABR for early-stage lung cancer across all age groups, which also support surgery as the preferred option when feasible, as outlined in current guidelines [15–17]. It is crucial for elderly patients and their healthcare providers to carefully weigh the potential benefits of surgery against the risks. A thorough discussion should consider the patient’s overall health, comorbidities, and personal preferences. Multidisciplinary teams, including thoracic surgeons, oncologists, and geriatric specialists, can provide valuable insights and help tailor the treatment plan to each patient’s needs.

However, the advantages of surgery may be exaggerated. Currently, SABR is recommended as the standard radiotherapy modality for managing inoperable early lung cancer [4]. It is important to note that not all instances of radiotherapy in the SEER database involved the use of SABR. Given the significant survival disparities between conventional fractionated radiotherapy and SABR [10], as evidenced in previous studies, this discrepancy may have contributed to the observed survival advantages of surgery over radiotherapy in our investigation. For patients with operable early-stage NSCLC, some prospective trials have shown that long-term survival after SABR is noninferior to that after lobectomy [18, 19]. A single-arm, prospective trial compared the SABR cohort to a PSM contemporary cohort treated with VATS lobectomy and mediastinal lymph node dissection in operable stage IA NSCLC patients. The comparison revealed a 3-year OS rate of 91% for both treatment groups and 5-year CSS rates of 87% for SABR and 84% for VATS lobectomy [18]. A 10-year follow-up of 112 patients with T1a-bN0M0 NSCLC who received SBRT compared with 1003 patients who underwent surgery revealed that the 10-year lung CSS was not significantly different (88% for SBRT versus 90% for surgery, *P* = 0.55). Although the 10-year OS was significantly different (45% versus 75%, *P* < 0.0001), after propensity score matching, the OS was no longer different among the cohorts (*P* = 0.74) [19].

Lobectomy is the standard surgical procedure for early-stage NSCLC. In select patients, sublobar resection, including segmentectomy or wedge resection, is appropriate for (1) individuals who are not eligible for lobectomy and (2) those with a peripheral nodule 2 cm or less with very low-risk features [4]. One multicenter, noninferiority, phase 3 trial revealed that in NSCLC patients with tumors ≤ 2 cm in size, sublobar resection was not inferior to lobectomy with respect to disease-free survival, with 5-year disease-free survival rates of 63.6% after sublobar resection and 64.1% after lobar resection [20]. Within our surgical cohort, lobectomy remained the predominant surgical approach, with a utilization rate of 65.8%, followed by wedge resection at 23.0% and segmentectomy at 7.7%. There was no significant difference in 1-year OS among the different surgical procedures, while long-term OS indicated superior outcomes for lobectomy compared with segmentectomy and wedge resection (3-year OS 75.2%, 71.2% and 65.0%, respectively). This may be attributed to the lower metastasis rate of lobectomy, and patients who undergo lobectomy may have better cardiopulmonary reserve than those who undergo sublobar resection. In addition, a larger tumor tended to favor lobectomy in 43.8% of patients in the 0–1 cm group and 84.5% of patients in the 4–5 cm group, which is consistent with current clinical practice.

Our study had several limitations. First, its retrospective nature increased potential for selection and information bias. Second, the SEER database lacks critical clinical details such as patient comorbidities and performance status which could significantly influence treatment outcomes and survival. Patients with fewer comorbidities and better performance status are more likely to undergo surgery rather than radiotherapy, potentially exaggerating the observed survival benefits of surgery. Although we attempted to minimize this bias by comparing patients who were recommended for surgery but refused and opted for radiotherapy instead, the impact of these unmeasured factors remains a concern. Third, the database does not distinguish between conventional radiotherapy and SABR. This limitation could underestimate SABR’s effectiveness when compared to surgery. Fourth, the database does not capture details on recurrence rates, treatment adherence, or long-term toxicities, which are critical in assessing the full impact of different treatment strategies. Future research utilizing the SEER-Medicare database or prospective clinical studies could help address these gaps and provide a more robust analysis.

Although our study primarily focused on survival outcomes, it is important to acknowledge that, for older patients, quality of life and patient preferences are equally critical factors in clinical decision-making. Surgery, while potentially offering superior long-term survival, is associated with significant perioperative risks and recovery burdens, which may not align with the priorities of all elderly patients. In contrast, radiotherapy, particularly SABR, provides a non-invasive alternative with fewer acute complications, making it an attractive option for those who prioritize maintaining independence and minimizing physical stress during treatment. Clinicians should carefully balance survival outcomes with patient-centered considerations, including quality of life (QoL) and individual treatment goals. Integrating tools such as QoL assessments and shared decision-making aids into clinical workflows could help personalize treatment strategies and optimize outcomes for this vulnerable population. Future research should integrate patient-reported outcomes to better understand how different treatment modalities impact physical, emotional, and social well-being in this population.

## Conclusion

The proportion of early-stage NSCLC patients aged 80 and older increased from 2011 to 2017 to 2018–2020. Radiotherapy is being increasingly used to treat early-stage NSCLC in octogenarians and older patients. However, for patients who may be candidates for both surgery and radiotherapy treatment, the long-term OS favors surgical management. Our findings support the current clinical guidelines recommending surgery as the preferred treatment approach for these patients. While survival is crucial, quality of life and patient are equally important in treatment decisions, underscoring the need for future research on the broader impacts of treatment on older patients’ well-being.

## Electronic supplementary material

Below is the link to the electronic supplementary material.

Supplementary Material 1

## Data Availability

No datasets were generated or analysed during the current study.

## Abbreviations

SEER: Surveillance, Epidemiology, and End Results database
NSCLC: Non-small cell lung cancer
RT: Radiotherapy
SABR: Stereotactic ablative radiotherapy
LNE: Lymph node examination
OS: Overall survival
CSS: Cancer-specific survival
mOS: Median OS
PSM: Propensity score-matching
QoL: Quality of life
