# Supplementary material for: Comparison of surgical and radiotherapy outcomes in octogenarians with early-stage non-small cell lung cancer: a SEER database retrospective cohort study
Source: Aging Clin Exp Res. 2025 Feb 27;37(1):53. doi: 10.1007/s40520-025-02948-2 (PMC11865165; doi:10.1007/s40520-025-02948-2)
Supplement: Supplementary file 1 — Supplementary Material 1 [file 40520_2025_2948_MOESM1_ESM.docx]

eFig. 1 Comparison of OS and CSS between surgery and radiotherapy by tumor size


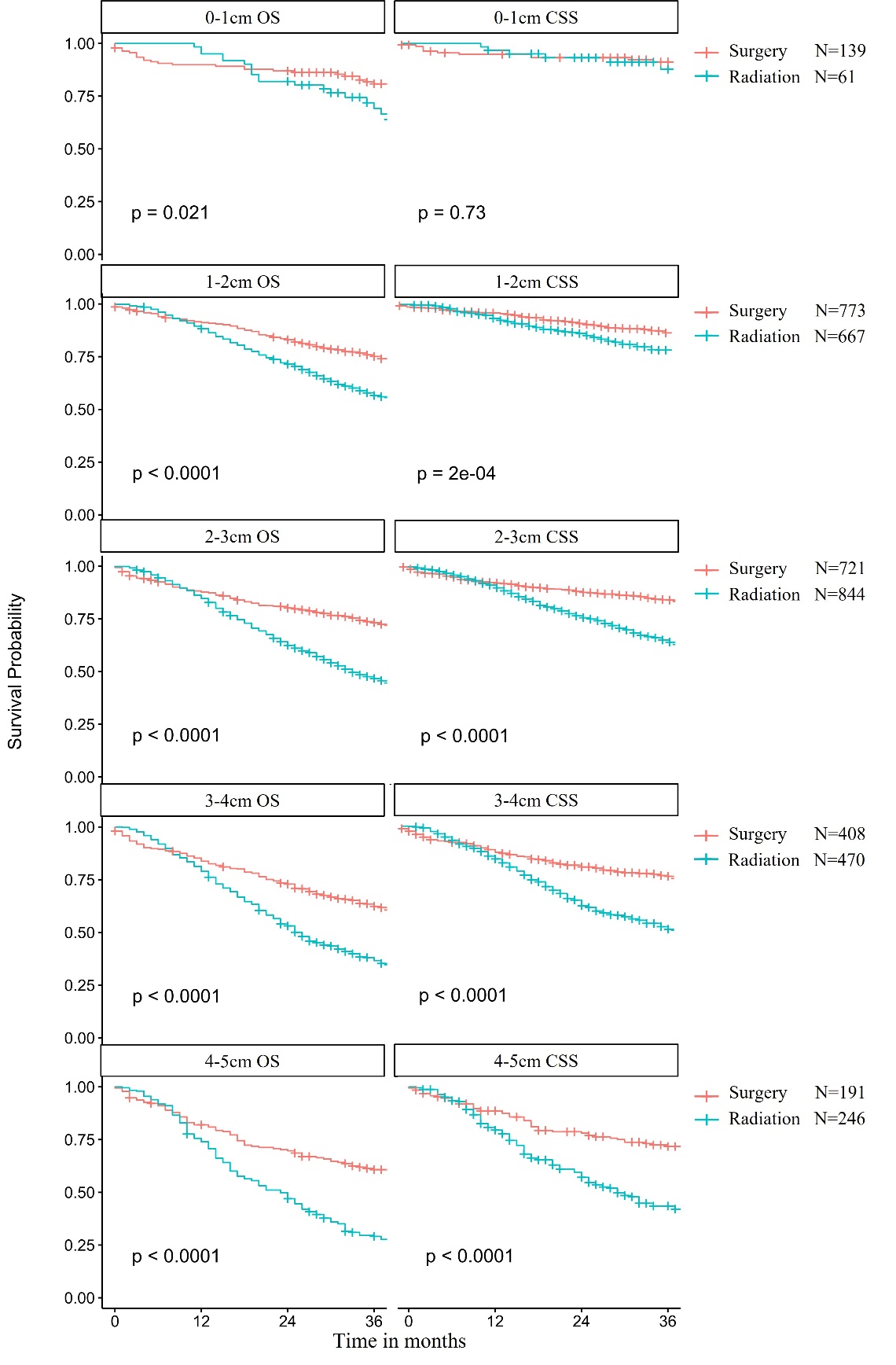


| eTable 1 Characteristics of patients in the surgery and radiotherapy groups before and after PSM | | | | | | | |
| --- | --- | --- | --- | --- | --- | --- | --- |
| Variable | Before PSM | | |  | After PSM | | |
|  | Surgery | Radiation | *P* value |  | Surgery | Radiation | *P* value |
| **All participants** | 2065 (100.0%) | 1217(100.0%) |  |  | 1217 (100.0%) | 1217 (100.0%) |  |
| **Gender** |  |  | 0.479 |  |  |  | 1.000 |
| Male | 860 (41.6%) | 523 (43.0%) |  |  | 522 (42.9%) | 523 (43.0%) |  |
| Female | 1205 (58.4%) | 694 (57.0%) |  |  | 695 (57.1%) | 694 (57.0%) |  |
| **Age** |  |  | <0.001 |  |  |  | <0.001* |
| 80-89 | 2007 (97.2%) | 1075 (88.3%) |  |  | 1159 (95.2%) | 1075 (88.3%) |  |
| ≥90 | 58 (2.8%) | 142 (11.7%) |  |  | 58 (4.8%) | 142 (11.7%) |  |
| **Race** |  |  | <0.001 |  |  |  | 0.428 |
| White | 1813 (87.8%) | 1051 (86.4%) |  |  | 1059 (87.0%) | 1051 (86.4%) |  |
| Black | 78 (3.8%) | 70 (5.8%) |  |  | 61 (5.0%) | 70 (5.8%) |  |
| Asian or Pacific Islander | 170 (8.2%) | 87 (7.1%) |  |  | 93 (7.6%) | 87 (7.1%) |  |
| Others | 4 (0.2%) | 9 (0.7%) |  |  | 4 (0.3%) | 9 (0.7%) |  |
| **Rural-Urban** |  |  | 0.112 |  |  |  | 0.743 |
| Urban | 1876 (90.8%) | 1084 (89.1%) |  |  | 1090 (89.6%) | 1084 (89.1%) |  |
| Rural | 189 (9.2%) | 133 (10.9%) |  |  | 127 (10.4%) | 133 (10.9%) |  |
| **Median household income** |  |  | 0.155 |  |  |  | 0.533 |
| $75,000+ | 844 (40.9%) | 466 (38.3%) |  |  | 482 (39.6%) | 466 (38.3%) |  |
| <$75,000 | 1221 (59.1%) | 751 (61.7%) |  |  | 735 (60.4%) | 751 (61.7%) |  |
| **Years of diagnosis** |  |  | <0.001 |  |  |  | 0.433 |
| 2011-2012 | 534 (25.9%) | 241 (19.8%) |  |  | 212 (17.4%) | 241 (19.8%) |  |
| 2013-2014 | 515 (24.9%) | 276 (22.7%) |  |  | 295 (24.2%) | 276 (22.7%) |  |
| 2015-2016 | 524 (25.4%) | 347 (28.5%) |  |  | 360 (29.6%) | 347 (28.5%) |  |
| 2017-2018 | 492 (23.8%) | 353 (29.0%) |  |  | 350 (28.8%) | 353 (29.0%) |  |
| **Histology** |  |  | <0.001 |  |  |  | 0.585 |
| ACA | 1363 (66.0%) | 665 (54.6%) |  |  | 689 (56.6%) | 665 (54.6%) |  |
| SCC | 541 (26.2%) | 476 (39.1%) |  |  | 459 (37.7%) | 476 (39.1%) |  |
| Other | 161 (7.8%) | 76 (6.2%) |  |  | 69 (5.7%) | 76 (6.2%) |  |
| **Grade** |  |  | <0.001 |  |  |  | 0.834 |
| Grade I | 514 (24.9%) | 256 (21.0%) |  |  | 256 (21.0%) | 256 (21.0%) |  |
| Grade II | 1006 (48.7%) | 499 (41.0%) |  |  | 518 (42.6%) | 499 (41.0%) |  |
| Grade III | 528 (25.6%) | 454 (37.3%) |  |  | 434 (35.7%) | 454 (37.3%) |  |
| Grade IV | 17 (0.8%) | 8 (0.7%) |  |  | 9 (0.7%) | 8 (0.7%) |  |
| **Tumor size** |  |  | <0.001 |  |  |  | 0.977 |
| 0-1cm | 116 (5.6%) | 28 (2.3%) |  |  | 30 (2.5%) | 28 (2.3%) |  |
| 1-2cm | 714 (34.6%) | 344 (28.3%) |  |  | 337 (27.7%) | 344 (28.3%) |  |
| 2-3cm | 666 (32.3%) | 442 (36.3%) |  |  | 437 (35.9%) | 442 (36.3%) |  |
| 3-4cm | 388 (18.8%) | 268 (22.0%) |  |  | 269 (22.1%) | 268 (22.0%) |  |
| 4-5cm | 181 (8.8%) | 135 (11.1%) |  |  | 144 (11.8%) | 135 (11.1%) |  |
| *The *P* value for age was significant; however, the standardized mean difference (SMD) was 0.0690 (<0.1),  indicating sufficient matching. | | | | | | | |

| eTable 2 Comparison of OS and CSS between surgery radiotherapy by tumor size | | | | | | | | | |
| --- | --- | --- | --- | --- | --- | --- | --- | --- | --- |
| Tumor size (cm) | Treatment | N | Median Survival (range) (mo) | OS | |  | CSS | |  |
|  |  |  |  | 1-Year Survival | 3-Year Survival | *P* | 1-Year Survival | 3-Year Survival | *P* |
| 0-1 | Surgery | 139 | 84(71-NA) | 89.87% | 80.82% | 0.021 | 94.77% | 91.23% | 0.73 |
|  | Radiation | 61 | 65(39-NA) | 95.08% | 69.20% |  | 96.72% | 87.73% |  |
| 1-2 | Surgery | 773 | 71(68-79) | 91.15% | 75.07% | <0.001 | 95.88% | 87.28% | <0.001 |
|  | Radiation | 667 | 45(40-50) | 88.29% | 56.57% |  | 94.74% | 78.25% |  |
| 2-3 | Surgery | 721 | 69(62-74) | 87.74% | 73.23% | <0.001 | 92.55% | 84.41% | <0.001 |
|  | Radiation | 844 | 33(31-37) | 84.67% | 46.72% |  | 91.29% | 65.26% |  |
| 3-4 | Surgery | 408 | 52(44-62) | 83.77% | 62.35% | <0.001 | 88.87% | 76.54% | <0.001 |
|  | Radiation | 470 | 26(23-28) | 78.94% | 36.66% |  | 85.82% | 52.29% |  |
| 4-5 | Surgery | 191 | 49(43-61) | 82.01% | 60.74% | <0.001 | 88.61% | 71.73% | <0.001 |
|  | Radiation | 246 | 23(19-26) | 73.96% | 29.12% |  | 79.48% | 43.45% |  |

| eTable 3 Comparison of OS and CSS between surgery without LNE and radiotherapy by tumor size | | | | | | | | | |
| --- | --- | --- | --- | --- | --- | --- | --- | --- | --- |
|  | Before PSM | | | |  | After PSM | | | |
|  | N | | *P* value | |  | N | | *P* value | |
| Size(cm) | Radiation | Surgery | OS | CSS |  | Radiation | Surgery | OS | CSS |
| 0-1 | 61 | 70 | 0.19 | 0.94 |  | 25 | 70 | 0.12 | 0.9 |
| 1-2 | 667 | 351 | <0.001 | 0.065 |  | 178 | 351 | <0.001 | 0.011 |
| 2-3 | 844 | 234 | <0.001 | 0.00068 |  | 159 | 234 | <0.001 | 0.021 |
| 3-4 | 470 | 117 | 0.0015 | 0.023 |  | 74 | 117 | 0.0062 | 0.037 |
| 4-5 | 246 | 42 | 0.00061 | 0.0037 |  | 37 | 42 | 0.028 | 0.058 |

| eTable 4 Comparison of OS and CSS between surgery and radiation (refused surgery) by tumor size | | | | | | | | | |
| --- | --- | --- | --- | --- | --- | --- | --- | --- | --- |
|  | Before PSM | | | |  | After PSM | | | |
|  | N | | *P* value | |  | N | | *P* value | |
| Size(cm) | Radiation | Surgery | OS | CSS |  | Radiation | Surgery | OS | CSS |
| 0-1 | 2 | 139 | The sample size is too small to analyze | | | | | | |
| 1-2 | 48 | 773 | 0.057 | 0.45 |  | 48 | 48 | 0.11 | 0.73 |
| 2-3 | 62 | 721 | 0.00024 | 0.00088 |  | 62 | 62 | 0.012 | 0.032 |
| 3-4 | 43 | 408 | 0.01 | 0.00017 |  | 43 | 43 | 0.0078 | 0.0011 |
| 4-5 | 21 | 191 | 0.096 | 0.53 |  | 21 | 21 | 0.51 | 0.95 |
